# EBA-related body-preferring responses in the occipitotemporal cortex measured with functional near-infrared spectroscopy

**DOI:** 10.3389/fnhum.2026.1914488

**Published:** 2026-09-16

**Authors:** Yuanchun Liu, Qiuya Zhao, Jiayu Wu, Ping Yang, Yuannong Ye, Min Wang

**Affiliations:** 1 School of Medical Humanities, Guizhou Medical University, Guiyang, China; 2 School of Biology and Engineering, Guizhou Medical University, Guiyang, China; 3 School of Psychology, Guizhou Normal University, Guiyang, Guizhou, China

**Keywords:** body-part selectivity, body-related visual processing, EBA, Extrastriate body area, fNIRS, occipitotemporal cortex

## Abstract

**Background:**

The extrastriate body area (EBA) is involved in the visual processing of bodies and body parts, but most evidence for its response profile comes from fMRI. We examined whether fNIRS can detect body-preferring occipitotemporal responses consistent with EBA-related processing.

**Methods:**

Sixty university students passively viewed non-hand body parts, hands, and objects. HbO responses were estimated using a block-level general linear model, with the three conditions matched in temporal structure. For each participant, a leave-one-subject-out (LOSO) procedure used the other 59 participants to select left and right functional peak channels within anatomically guided EBA-related occipitotemporal channel pools. This reduced direct participant-level circularity but did not provide dataset-level independence or independently localized EBA ROIs. Preprocessing included linear detrending, temporal derivative distribution repair, and 0.01–0.08-Hz band-pass filtering. An additional ICA preprocessing step with a conservative noise identification threshold was applied to attenuate spatially widespread noise components.

**Results:**

In the primary analysis, both the body–object and body–hand contrasts were significant for the left and right LOSO-selected functional peak channels and the bilateral average after FDR correction. Following additional ICA preprocessing, both contrasts remained significant for the left functional peak channel and the bilateral average, but not for the right functional peak channel.

**Conclusion:**

Non-hand body parts elicited stronger HbO responses than objects and hands in posterior temporal–occipital cortex. The left-hemisphere and bilateral effects were significant in both analyses, whereas the right-sided effects were significant only in the primary analysis. Although the absence of short-separation measurements precluded complete separation of cortical and extracerebral contributions, the findings support the feasibility of measuring body-preferring occipitotemporal responses consistent with EBA-related processing using fNIRS; they do not establish direct anatomical localization of the EBA.

## 1 Introduction

Human bodies and body parts convey socially and behaviorally relevant visual information in everyday life. Body-related visual cues help observers recognize actions, infer others’ intentions, and interpret socially relevant signals such as gestures and emotional body expressions (de Gelr, 2006; Peelen and Downing, 2007). Consistent with this functional significance, the human visual system contains category-selective regions that are particularly sensitive to body-related stimuli (Downing et al., 2001; Peelen and Downing, 2007).

A central body-selective region is the extrastriate body area (EBA), a functional region located in the lateral occipitotemporal cortex (Pann et al., 2021; Rosenke et al., 2021). The EBA responds more strongly to images of bodies and body parts than to non-body objects (Downing et al., 2001; Peelen and Downing, 2007). Previous studies have implicated the EBA in the visual analysis of body form and body parts (Schwarzlose et al., 2005; Taylor et al., 2007). Causal evidence from transcranial magnetic stimulation further suggests that the EBA contributes to the visual processing of non-face body parts (Urgesi et al., 2004). In addition, neuropsychological evidence from lesion studies indicates that damage involving the EBA is associated with impaired recognition of body stimuli, whereas object recognition can remain relatively preserved (Moro et al., 2008). More recent neuroimaging work has further refined this account. Ultra-high-field fMRI encoding analyses indicate that body representations in the occipitotemporal cortex and EBA reflect a combination of low-level visual and postural features (Marrazzo et al., 2023). Converging fMRI and fMRI-guided transcranial magnetic stimulation evidence also suggests a causal contribution of the left EBA to visual sensitivity to face-to-face social interactions (Gandolfo et al., 2024). Together, these classic and recent findings support the EBA as a useful functional marker for body-related visual processing while emphasizing the diversity of information represented within body-selective occipitotemporal cortex.

Importantly, hand representations may not be identical to representations of other body parts. Hands are visually and functionally important because they are closely linked to action execution, object manipulation, and tool use (Knights et al., 2021). Previous fMRI evidence has shown that distributed response patterns in visual cortex can discriminate hands from other body-related categories such as torsos (Op de Beeck et al., 2010). More directly, Bracci et al. (2010) reported dissociable responses to hands and non-hand body parts in human extrastriate visual cortex. Specifically, they identified a hand-preferring region in the left lateral occipitotemporal cortex, centered in the lateral occipital sulcus. This region partially overlapped with the left EBA but was functionally and anatomically distinguishable from it; in most participants, the hand-preferring region was located anterior to the left EBA, whereas EBA was represented bilaterally. In contrast to this hand-preferring region, the EBA showed stronger responses to bodies and non-hand body parts than to hands. These findings suggest that the lateral occipitotemporal cortex contains partially distinct response profiles for hands and non-hand body parts.

Most evidence for the EBA and body-part selectivity has come from functional magnetic resonance imaging (fMRI). fMRI provides high spatial resolution and is well suited for identifying category-selective visual regions. However, fMRI is expensive, less portable, and requires participants to remain still inside a scanner. Functional near-infrared spectroscopy (fNIRS) provides a more flexible alternative by measuring task-related changes in oxygenated and deoxygenated hemoglobin from superficial cortical regions (Cutini et al., 2012; Ferrari and Quaresima, 2012; Pinti et al., 2020). This feature is relevant for EBA-related research because the EBA is located in the lateral occipitotemporal cortex, a region that is accessible to appropriately positioned occipitotemporal fNIRS probes. Consistent with this possibility, Ishizu et al. (2009) used near-infrared spectroscopy to measure activity in an occipitotemporal body-selective region during body-related tasks, and Shimada (2013) further reported that EBA-related activity during body-part and object viewing could be detected with fNIRS combined with 3D digitizer-based probe localization. More recently, Chai et al. (2024) demonstrated that fNIRS could detect category-selective object responses in the lateral occipital complex, although face-selective responses in the deeper fusiform face area were not reliably detected. This recent work highlights both the feasibility and the depth-related limitations of applying fNIRS to category-selective regions in the ventral visual pathway. Taken together, the available evidence suggests that near-infrared methods can capture category-preferring hemodynamic responses from accessible occipitotemporal cortex, although they do not provide the same spatial specificity as fMRI.

Despite these developments, direct fNIRS evidence for EBA-related body selectivity remains sparse compared with the extensive and increasingly detailed fMRI literature on body-selective visual cortex. Recent fNIRS work supports the feasibility of measuring some lateral ventral-visual targets, but it remains unclear whether occipitotemporal fNIRS can distinguish responses to non-hand body parts from responses to both objects and hands. Further validation of EBA-related fNIRS measurements is therefore needed. Moreover, the lateral occipitotemporal location of the EBA poses methodological challenges for fNIRS because signal quality depends strongly on probe placement, cortical depth, and individual anatomical variability. Another important issue is spatial specificity. Because fNIRS has lower spatial resolution than fMRI, it cannot precisely separate adjacent category-selective regions within the lateral occipitotemporal cortex, including the EBA and nearby hand-preferring regions such as the left lateral occipital sulcus. Therefore, fNIRS findings should be described cautiously as EBA-related or EBA-like responses rather than as exact anatomical localization of the EBA itself.

The present study aimed to further evaluate whether fNIRS can detect body-preferring occipitotemporal responses consistent with EBA-related processing. We used a passive viewing task with non-hand body-part images, hand images, and object images, together with a targeted occipitotemporal fNIRS probe layout. The body-object contrast tested whether fNIRS could detect body-selective responses (non-hand body parts > objects), whereas the body-hand contrast tested whether EBA-related channels showed stronger responses to non-hand body parts than to hands. Based on previous fMRI and fNIRS evidence, we expected non-hand body-part images to elicit greater oxygenated hemoglobin responses than both object images and hand images in EBA-related occipitotemporal channels.

## 2 Materials and methods

### 2.1 Participants and ethical approval

Sixty university students were included in the final analysis. The mean age of the participants was 21.21 years (SD = 2.41), and 45 participants were female. All participants had normal or corrected-to-normal vision. Participants received compensation for their participation. Written informed consent was obtained from all participants before the experiment. The study was approved by the Ethics Committee of Guizhou Medical University (Approval No. 342/2025) and was conducted in accordance with the Declaration of Helsinki.

### 2.2 fNIRS recording system and probe layout

Cortical hemodynamic activity was recorded using a LIGHTNIRS system (Shimadzu Corporation, Kyoto, Japan; wavelengths: 780, 805, and 830 nm). The source-detector separation for the measurement channels was 3 cm, and the sampling rate was 41.7 Hz.

Before formal data collection, a separate montage-positioning test was conducted to guide the arrangement of the bilateral occipitotemporal probe arrays. Published stereotaxic coordinates of the EBA and body-selective activations in the lateral occipitotemporal cortex were visualized in Montreal Neurological Institute (MNI) space to define approximate bilateral anatomical target clusters (Downing et al., 2001; Peelen and Downing, 2005; Schwarzlose et al., 2005; Bracci et al., 2010). Within the constraints imposed by the fixed optode-array geometry and the 3-cm source–detector separation, the optodes were arranged to provide broad coverage around these coordinate clusters and the surrounding lateral occipitotemporal cortex. Anatomical landmarks and optode positions obtained during this pre-experimental positioning test were recorded using a three-dimensional positioning procedure and probabilistically mapped into MNI space. BrainNet Viewer (Xia et al., 2013) was used to inspect the expected cortical coverage of the resulting montage. The same standardized montage-placement procedure was subsequently used in the formal experiment. This pre-experimental procedure was intended to guide montage design and expected anatomical coverage; participant-specific three-dimensional registration and an independent functional EBA localizer were not obtained for the formal sample.

The final standardized montage consisted of two optode arrays, one over each hemisphere, and yielded 20 measurement channels. The midpoint between each light emitter and detector was used as the approximate location of each measurement channel. All usable channels within each hemisphere-specific array constituted the predefined left and right occipitotemporal channel pools, respectively. These channel pools were anatomically guided but were not treated as independently localized EBA ROIs. The channel distribution and optode arrangement are shown in Figure 1, and the estimated MNI coordinates of the 20 channels obtained from the pre-experimental positioning test are provided in Supplementary Table S1.

**Figure 1:**
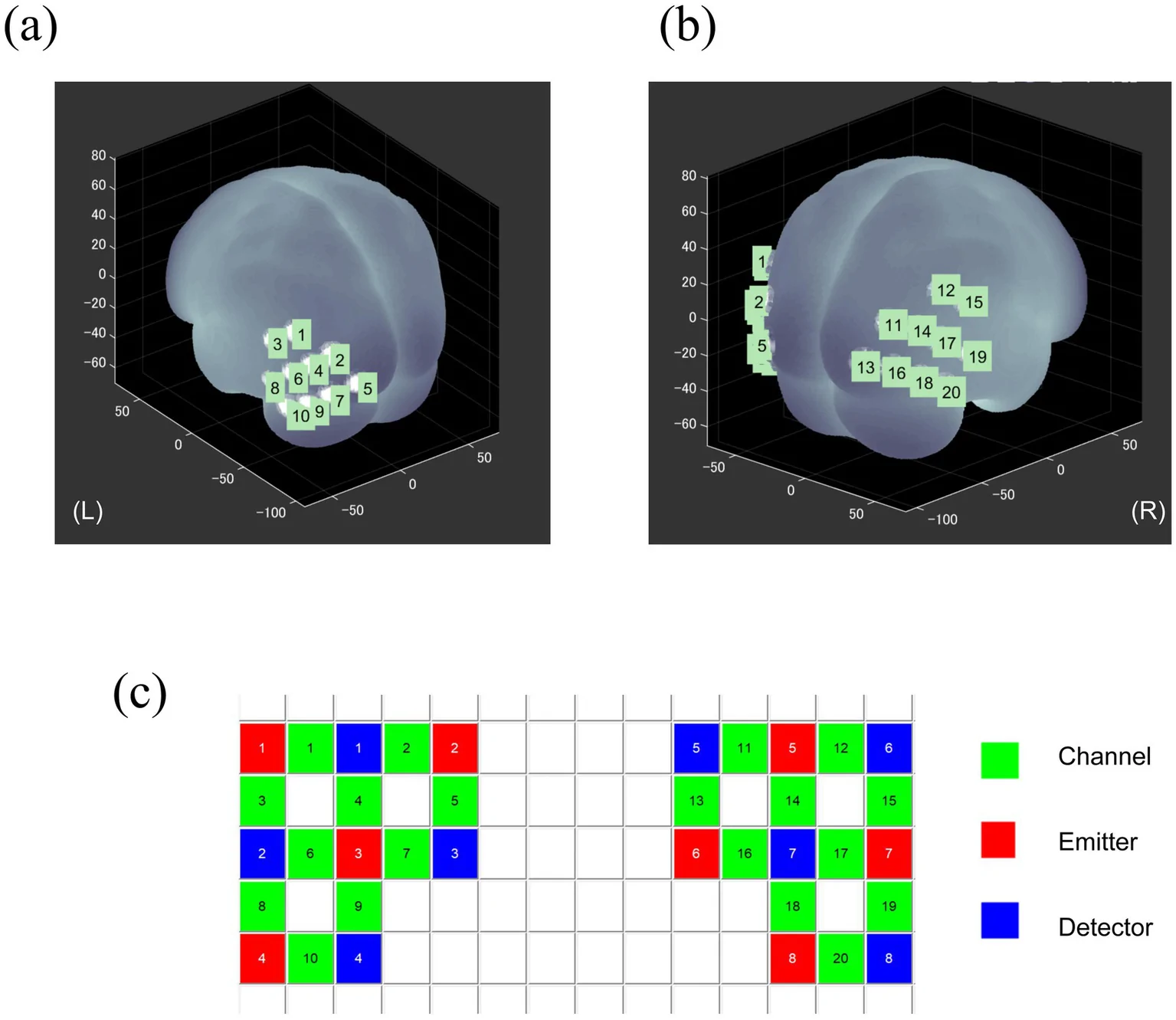
Arrangement of the bilateral occipitotemporal fNIRS montage. **(a)** Left-hemisphere channels and **(b)** Right-hemisphere channels displayed on the standard brain template. **(c)** Optode arrangement, with green squares indicating measurement channels, red squares indicating light emitters, and blue squares indicating detectors. The displayed channel locations reflect the expected coverage derived from a separate pre-experimental montage-positioning test rather than participant-specific registration or direct anatomical localization of the EBA. Estimated MNI coordinates are provided in Supplementary Table S1.

### 2.3 Stimuli and experimental task

The task was programmed using E‑Prime 3.0 and presented on a 27‑inch monitor with a resolution of 1,920 × 1,080 pixels. Participants sat approximately 60 cm from the monitor, and the center of the monitor was aligned with the participant’s eye level.

Three stimulus categories were used: non-hand body parts, hands, and objects (Figure 2c). Each category included 15 images. The non-hand body-part and object images used in the experiment were selected from the stimulus materials kindly provided by the authors of Downing et al. (2001), whereas the hand images were photographed by the authors of the present study. Non-hand body-part images and hand images were treated as separate categories, allowing the present study to test whether EBA-related channels responded more strongly to non-hand body parts than to hands.

**Figure 2:**
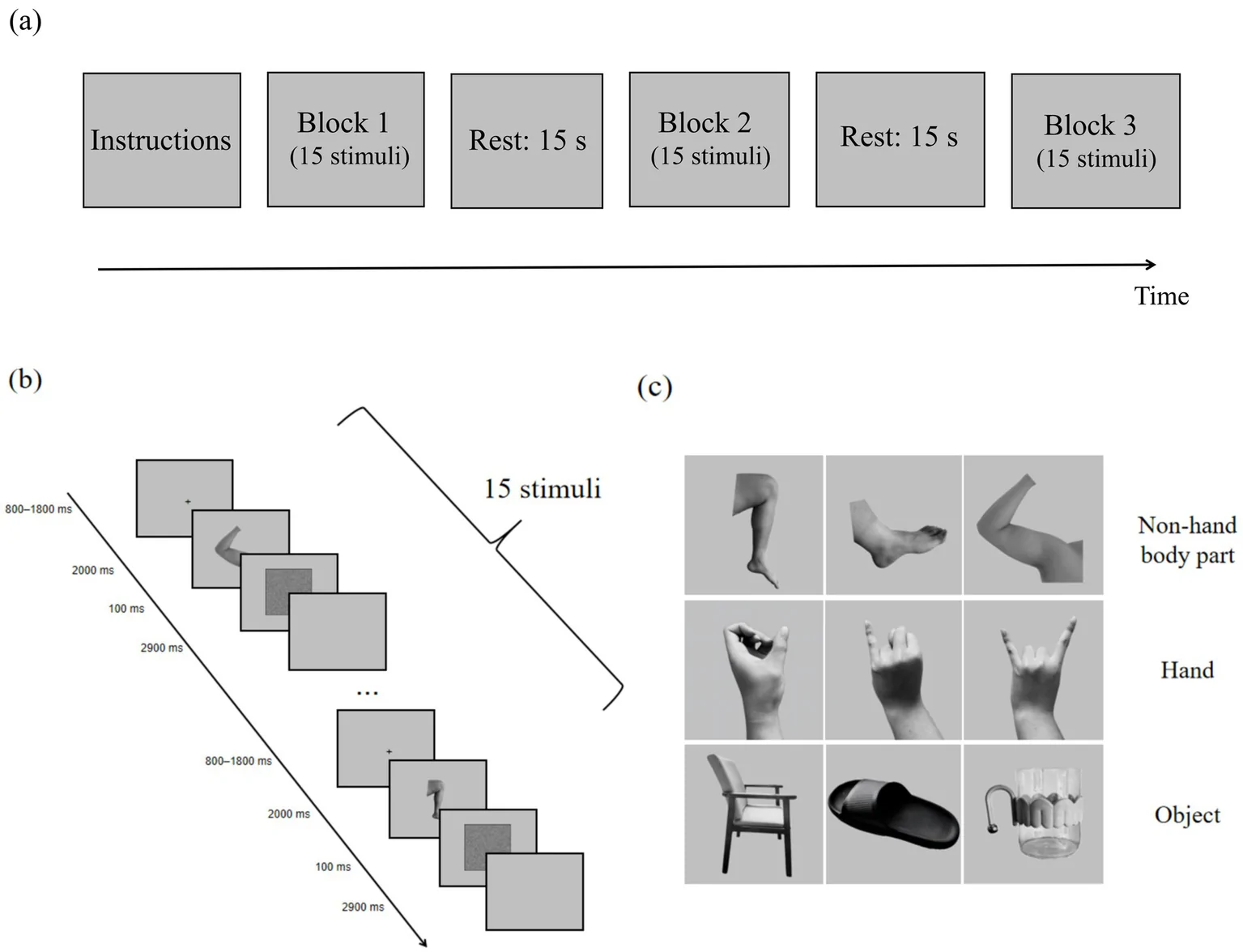
Schematic illustration of the experimental procedure and stimulus materials. **(a)** Overall block sequence. The task began with an instruction screen and consisted of three category blocks. Each block contained 15 stimuli, and a 15-s rest interval was inserted between adjacent blocks. The three category blocks corresponded to non-hand body parts, hands, and objects, and their order was randomized across participants. **(b)** Trial structure within each category block. Each trial began with a fixation cross presented for 800–1,800 ms, followed by image presentation for 2,000 ms, a visual mask for 100 ms, and a blank-screen interval for 2,900 ms. This trial structure was repeated for 15 stimuli within each category block and was identical across the three stimulus categories. The example shown in this panel illustrates a non-hand body-part block. **(c)** Examples of the three stimulus categories: Non-hand body parts, hands, and objects. The images in panel **(c)** were newly photographed by the authors for illustrative purposes and were not used in the actual experiment; however, they are highly similar to the experimental stimuli in terms of visual features.

Participants completed a passive viewing task and were instructed to view the images attentively while minimizing head and body movements. No overt response was required. Stimuli were presented in a block design. Each stimulus category was presented in one block, resulting in three blocks in total (Figure 2a). Each trial began with a black fixation cross presented at the center of the monitor for a jittered duration of 800–1,800 ms, followed by one centrally presented image for 2,000 ms. After image presentation, a visual mask was presented for 100 ms, followed by a blank screen for 2,900 ms before the next trial began (Figure 2b). A 15‑s rest interval was inserted between adjacent blocks. The order of the three stimulus blocks was randomized across participants (See Figure 2).

### 2.4 fNIRS data preprocessing

Raw optical signals were converted into concentration changes in oxygenated hemoglobin (HbO) and deoxygenated hemoglobin (HbR). The present study focused on HbO as the primary dependent measure because HbO is commonly used in task-based fNIRS studies and is sensitive to stimulus-related cortical hemodynamic responses. Data preprocessing was performed using NIRS-KIT (Hou et al., 2021), following general reporting and preprocessing recommendations for fNIRS studies (Yücel et al., 2021).

First, slow drifts were removed using first-order linear detrending, motion artifacts were corrected using temporal derivative distribution repair (TDDR; Fishburn et al., 2019), and a 0.01–0.08 Hz band-pass filter was applied. The filtered HbO data were then submitted to participant-level temporal ICA using the NIRS-ICA toolbox (Zhao et al., 2021). Principal component analysis prewhitening retained components explaining 99% of the data variance, and second-order blind identification was performed using 100 time-delayed correlation matrices. Spatially global components were identified using the coefficient of spatial uniformity (CSU; Kohno et al., 2007). A fixed CSU cutoff of 1.9 was used for HbO, informed by the value reported by Kawai et al. (2026), and all components exceeding this cutoff were removed. The remaining components were visually inspected. Components showing task-synchronized, block-shaped waveforms were considered putative neuronal sources and retained, whereas those exhibiting sinusoidal oscillations or irregular waveforms were removed. The cleaned HbO data were reconstructed and then submitted to the same GLM analysis as the primary data.

### 2.5 Statistical analysis

A general linear model (GLM) was used to estimate HbO responses for each stimulus category in each channel (Tak and Ye, 2014). The design matrix included three block-level regressors corresponding to the non-hand body-part, hand, and object conditions. For each category, the onset was defined as the onset of the first image, and the duration spanned the complete block, including the intervening fixation, visual-mask, and blank-screen periods. Block-level modeling was used because the 15 trials within each category were presented consecutively and the fNIRS hemodynamic response evolves more slowly than individual trial events. The three conditions contained the same number of trials and followed the same temporal structure.

Beta estimates were extracted for each condition, and two planned contrasts were calculated: body–object (β_body − β_object) and body–hand (β_body − β_hand).

To reduce participant-level circularity in channel selection, a leave-one-subject-out (LOSO) procedure was used (Kriegeskorte et al., 2009). For each held-out participant, one functional peak channel was selected separately within the predefined left and right occipitotemporal channel pools using the average non-hand body-part responses of the other 59 participants. The selected channels were then used to extract contrast values from the held-out participant, and the left and right values were averaged to obtain a bilateral summary. Because the held-out participant’s own data did not contribute to channel selection, this procedure reduced participant-level circularity. However, channel selection and hypothesis testing were still based on the same cohort and task dataset, so the procedure did not provide dataset-level independence. The selected channels should therefore be interpreted as cross-validated functional peak channels in the present dataset rather than independently localized EBA ROIs.

Statistical significance was evaluated using six prespecified one-sample t-tests (B–O and B–H contrasts for the left and right functional peak channels and the bilateral average). For the primary and ICA-preprocessed data separately, the Benjamini–Hochberg FDR procedure was applied across the six tests, with FDR controlled at *q* = 0.05. Mean contrast values, standard deviations, two-sided 95% confidence intervals, and Cohen’s d_z_ were calculated for each contrast and regional summary. LOSO peak channels were reselected using the ICA-preprocessed data rather than carried forward from the primary analysis.

## 3 Results

### 3.1 Body-object contrast

The body–object contrast tested whether non-hand body-part blocks elicited stronger HbO responses than object blocks at the LOSO-selected functional peak channels. Positive contrast values indicated greater responses to non-hand body-part blocks relative to object blocks. One-sample *t*-tests showed a significant positive body–object effect for the left LOSO-selected functional peak channel, *t*(59) = 2.913, p_FDR_ = 0.005, d_z_ = 0.38. A significant positive effect was also observed for the right LOSO-selected functional peak channel, *t*(59) = 1.754, p_FDR_ = 0.042, d_z_ = 0.23. The bilateral average likewise showed a significant body-object effect, *t*(59) = 2.761, p_FDR_ = 0.006, d_z_ = 0.36.

For visualization, channel-wise t values for the body-object contrast were projected onto the cortical surface using BrainNet Viewer (Figure 3a). These spatial maps were intended to illustrate the distribution of the body–object effect across the sampled EBA-related occipitotemporal channels rather than to provide independent anatomical localization. Together, these results indicate that non-hand body-part blocks elicited greater HbO responses than object blocks at the LOSO-selected functional peak channels within the EBA-related occipitotemporal pools. This response difference is consistent with EBA-related body-preferring processing but does not constitute direct anatomical localization of the EBA.

**Figure 3:**
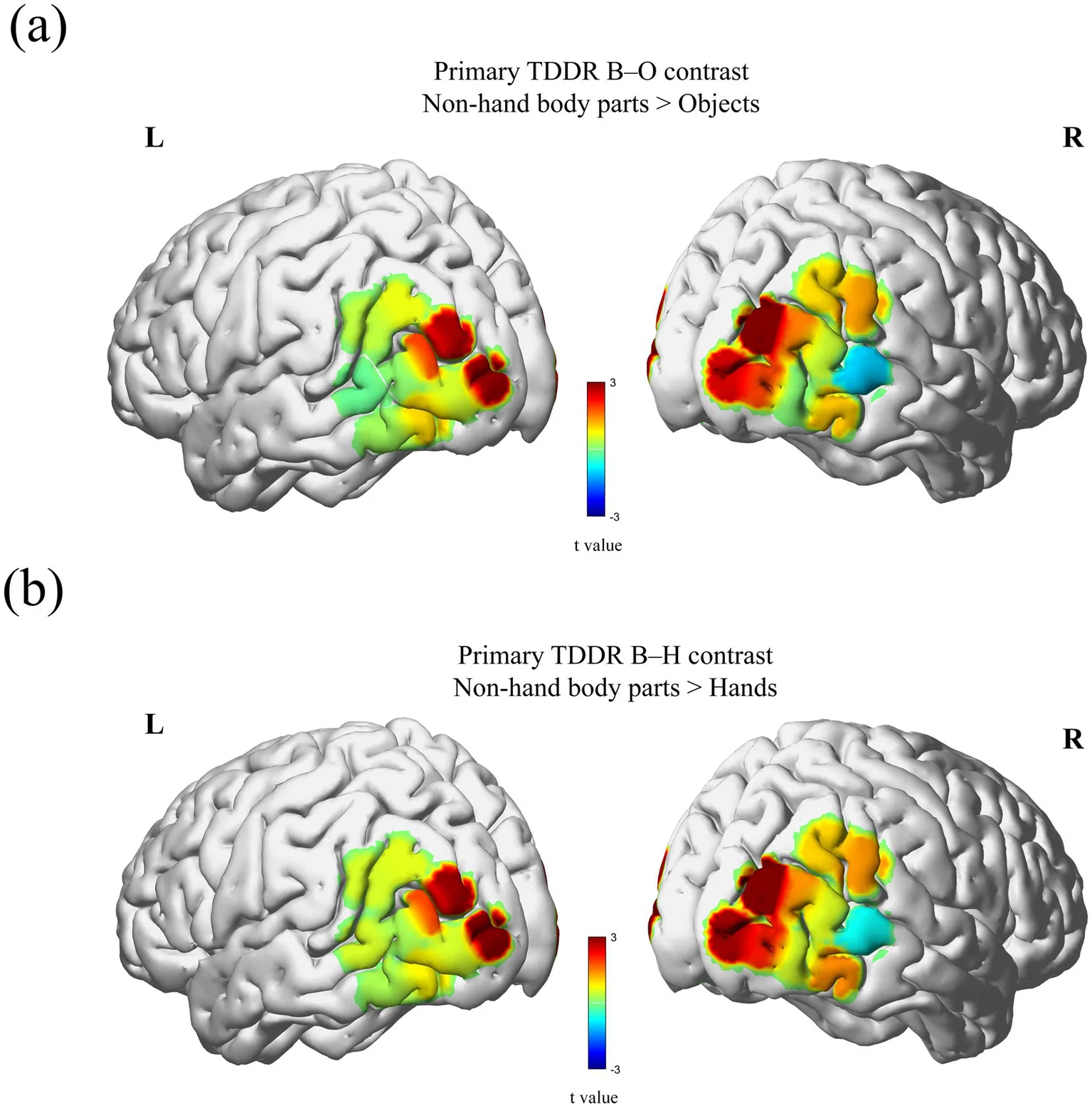
Primary TDDR channel-wise *t*-value maps for **(A)** the non-hand body part–object (B–O) contrast and **(B)** the non-hand body part–hand (B–H) contrast. L and R indicate the left and right hemispheres, respectively. The color scale represents channel-wise *t* values. These channel-level maps descriptively illustrate the distribution of functional responses across the sampled occipitotemporal channels and should not be interpreted as direct anatomical localization of the EBA or as an estimate of the precise spatial extent of EBA activity. Inferential conclusions are based on the prespecified LOSO regional tests with FDR correction.

### 3.2 Body-hand contrast

The body–hand contrast tested whether the LOSO-selected functional peak channels showed stronger HbO responses to non-hand body-part blocks than to hand blocks. Positive contrast values indicated greater responses to non-hand body-part blocks relative to hand blocks. One-sample t-tests showed a significant positive body–hand effect for the left LOSO-selected functional peak channel, *t*(59) = 3.188, p_FDR_ = 0.005, d_z_ = 0.41. A significant positive effect was also observed for the right LOSO-selected functional peak channel, *t*(59) = 2.092, p_FDR_ = 0.025, d_z_ = 0.27. The bilateral average likewise showed a significant body-hand effect, *t*(59) = 3.088, p_FDR_ = 0.005, d_z_ = 0.40.

Channel-wise t values for the body-hand contrast were projected onto the cortical surface using the same BrainNet Viewer procedure (Figure 3b). These results indicate that, within the LOSO-selected functional peak channels, non-hand body-part blocks elicited greater HbO responses than hand blocks. This response pattern is consistent with an EBA-related body-preferring profile. However, the B–H contrast reflects a relative response difference between the two stimulus conditions and does not independently localize or anatomically distinguish the EBA from hand-preferring cortical regions.

The inferential results and descriptive effect estimates, including mean contrast values, standard deviations, and two-sided 95% confidence intervals, are summarized in Table 1.

**Table 1:** Primary TDDR results and descriptive effect estimates for the B–O and B–H contrasts.

Contrast	Region	Mean (SD)	95% CI	t(df)	p_FDR_	d_z_
B–O	Left	0.002672 (0.007105)	[0.000837, 0.004507]	2.913 (59)	0.005	0.38
B–O	Right	0.001194 (0.005273)	[−0.000168, 0.002556]	1.754 (59)	0.042	0.23
B–O	Bilateral	0.001933 (0.005423)	[0.000532, 0.003334]	2.761 (59)	0.006	0.36
B–H	Left	0.002821 (0.006852)	[0.001051, 0.004591]	3.188 (59)	0.005	0.41
B–H	Right	0.001419 (0.005254)	[0.000062, 0.002776]	2.092 (59)	0.025	0.27
B–H	Bilateral	0.002120 (0.005318)	[0.000746, 0.003494]	3.088 (59)	0.005	0.40

B–O, body–object contrast; B–H, body–hand contrast. Mean and SD were calculated across the LOSO-extracted participant-level contrast values. The reported 95% CIs are conventional two-sided intervals calculated using the t distribution. p_FDR_ values are Benjamini–Hochberg FDR-adjusted *p* values across the six prespecified one-sample t-tests. d_z_ represents Cohen’s d_z_ for one-sample t-tests.

### 3.3 Results after ICA preprocessing

After ICA preprocessing using a fixed CSU cutoff of 1.9, both the B–O and B–H contrasts remained significant for the left functional peak channel and the bilateral average (all p_FDR_ ≤ 0.024), but not for the right functional peak channel (both p_FDR_ ≥ 0.095; Table 2). Peak-channel selections for the primary and ICA-preprocessed data are summarized in Table 3, and Figure 4 displays the corresponding ICA-preprocessed channel-wise *t*-value maps.

**Table 2:** Results and descriptive effect estimates for the B–O and B–H contrasts after ICA preprocessing.

Contrast	Region	Mean (SD)	95% CI	t(df)	p_FDR_	d_z_
B–O	Left	0.002234 (0.007004)	[0.000425, 0.004043]	2.471 (59)	0.016	0.32
B–O	Right	0.000684 (0.004800)	[−0.000557, 0.001925]	1.104 (59)	0.137	0.14
B–O	Bilateral	0.001459 (0.005148)	[0.000128, 0.002790]	2.195 (59)	0.024	0.28
B–H	Left	0.002339 (0.006756)	[0.000594, 0.004084]	2.682 (59)	0.016	0.35
B–H	Right	0.000872 (0.004727)	[−0.000349, 0.002093]	1.428 (59)	0.095	0.18
B–H	Bilateral	0.001605 (0.005019)	[0.000309, 0.002901]	2.478 (59)	0.016	0.32

B–O, body–object contrast; B–H, body–hand contrast. Significant FDR-corrected *p*-values are indicated in the table. ICA preprocessing was applied to HbO data.

**Table 3:** Distribution of peak-channel selections across the 60 LOSO folds in the primary and ICA-preprocessed data.

Analysis	Hemisphere	Selected peak channel	LOSO folds, *n*	Percentage (%)
Primary	Left	5	60	100
Primary	Right	11	52	86.7
Primary	Right	13	8	13.3
ICA-preprocessed	Left	5	60	100
ICA-preprocessed	Right	11	9	15
ICA-preprocessed	Right	13	51	85

Each LOSO fold corresponded to one left-out participant. Counts and percentages indicate the number and proportion of folds in which each channel was selected. Peak-channel selection was performed separately for the primary and ICA-preprocessed data using the corresponding data from the remaining 59 participants. The selected channels represent cross-validated functional peak channels in the present dataset and should not be interpreted as independently localized EBA ROIs.

**Figure 4:**
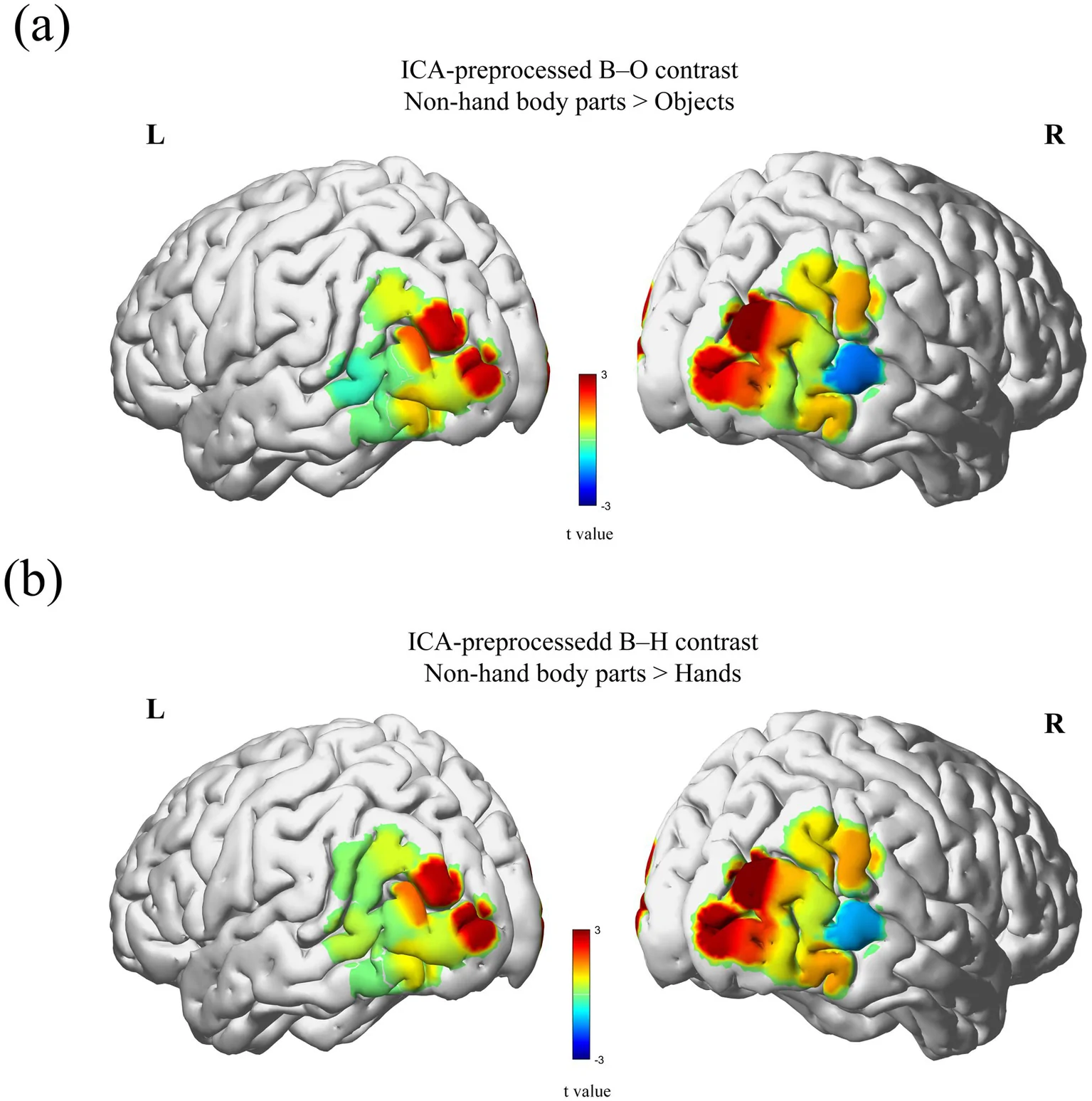
ICA-preprocessed channel-wise t-value maps for **(A)** the non-hand body part–object (B–O) contrast and **(B)** the non-hand body part–hand (B–H) contrast. L and R indicate the left and right hemispheres, respectively. The color scale represents channel-wise t values. These channel-level maps descriptively illustrate the distribution of functional responses across the sampled occipitotemporal channels after ICA preprocessing and should not be interpreted as direct anatomical localization of the EBA or as an estimate of the precise spatial extent of EBA activity. Inferential conclusions are based on the prespecified LOSO regional tests with FDR correction applied to the ICA-preprocessed data.

Across the primary analysis and ICA preprocessing, both contrasts converged for the left functional peak channel and the bilateral average. In contrast, the right-sided effects were significant in the primary analysis but not after ICA preprocessing.

## 4 Discussion

The present study examined whether occipitotemporal fNIRS could detect body-preferring HbO responses to non-hand body parts relative to objects and hands. In the primary analysis based on TDDR-preprocessed data, both the B–O and B–H contrasts were significant for the left and right LOSO-selected functional peak channels and the bilateral average. The same analysis was repeated after ICA preprocessing to assess preprocessing sensitivity. Overall, the findings are consistent with EBA-related body-preferring processing but should not be interpreted as direct anatomical localization of the EBA.

### 4.1 fNIRS detection of EBA-related body-preferring responses

The significant B–O effects indicate that non-hand body-part images elicited stronger HbO responses than object images within the LOSO-selected functional peak channels. This pattern is consistent with fMRI evidence showing stronger responses to bodies and body parts than to non-body objects in body-selective occipitotemporal cortex (Downing et al., 2001; Peelen and Downing, 2007). It also agrees with previous near-infrared studies showing that body-related activity can be measured from accessible occipitotemporal cortex (Ishizu et al., 2009; Shimada, 2013). Recent evidence that fNIRS can detect category-preferring responses in superficial regions of the ventral visual pathway further supports the feasibility of this approach (Chai et al., 2024).

After ICA preprocessing, both the B–O and B–H effects remained significant for the left functional peak channel and the bilateral average, whereas neither contrast remained significant for the right functional peak channel. The left and bilateral effects were therefore more consistent across the two preprocessing procedures, whereas the right-sided effects were more sensitive to preprocessing. However, the present data cannot determine whether this difference reflects greater physiological contamination on the right or a genuinely weaker underlying response in that hemisphere. These findings support the feasibility of using occipitotemporal fNIRS to measure body-preferring responses, but they do not establish hemispheric lateralization or direct anatomical localization of the EBA.

### 4.2 Body–hand contrast and the body-preferring response profile

The significant B–H effects indicate that non-hand body-part images elicited stronger HbO responses than hand images within the selected occipitotemporal channels. This finding is compatible with previous fMRI evidence that responses to hands and non-hand body parts are partly dissociable within lateral occipitotemporal cortex. Bracci et al. (2010) identified a hand-preferring region that partially overlapped with, but was functionally distinguishable from, the left EBA, whereas the EBA responded more strongly to bodies and non-hand body parts than to hands. The present B–H effect is therefore consistent with an EBA-related body-preferring response profile.

However, the B–H contrast represents a relative response difference between two stimulus categories. It does not independently localize either the EBA or hand-preferring cortex and cannot establish an anatomical dissociation between these regions. Accordingly, the present result should be interpreted as a stronger response to non-hand body parts than to hands within the sampled occipitotemporal cortex, rather than as evidence that fNIRS precisely separated EBA from adjacent hand-preferring regions.

### 4.3 Methodological considerations and spatial interpretation

The bilateral occipitotemporal montage was designed before formal data collection with reference to published coordinates of the EBA and body-selective occipitotemporal activations. Its expected cortical coverage was evaluated in a separate pre-experimental positioning test. This procedure provided an anatomical basis for defining the initial left and right occipitotemporal channel pools. However, the estimated channel coordinates did not represent participant-specific locations in the formal sample, and the LOSO peak channels were selected according to functional responses rather than their proximity to literature-derived EBA coordinates. The montage and channel-selection procedures therefore do not establish direct anatomical localization of the EBA.

The LOSO procedure excluded each held-out participant’s own data from the selection of the channels used to evaluate that participant, thereby reducing direct participant-level circularity. However, channel selection and hypothesis testing were based on the same cohort and task dataset, so the procedure did not provide dataset-level independence. The selected channels should therefore be interpreted as cross-validated functional peak channels in the present dataset, rather than as independently localized EBA ROIs. Notably, peak-channel selection was stable across LOSO folds for both the primary and ICA-preprocessed data (Table 3), indicating that the identity of the selected channels was not strongly affected by the exclusion of any single participant.

The use of one peak channel per hemisphere provided a sensitivity-oriented functional summary and was consistent with previous fNIRS research on EBA-related processing (Shimada, 2013). This approach avoided averaging channels with different cortical coverage or measurement sensitivity. However, it was not assumed to be anatomically superior to a multi-channel ROI and may be sensitive to individual differences in probe placement and cortical anatomy. Confirmation using participant-specific channel registration and an independent functional localizer is therefore needed.

### 4.4 Limitations and future directions

Several limitations should be noted. First, participant-specific channel registration and an independent functional localizer were not available. Although LOSO reduced participant-level circularity, channel selection and testing were still based on the same dataset, and a single peak channel may not capture the full spatial extent of the response. Validation using an independent dataset or functional localizer is therefore needed.

Second, short-separation channels and concurrent physiological recordings were unavailable, limiting the separation of cortical and extracerebral signals. ICA provided an additional sensitivity check but could not fully resolve this limitation. Future studies should include these measurements.

Third, the choice of CSU cutoff in ICA-based denoising involves a degree of subjectivity. The present study adopted a fixed threshold of 1.9 for HbO based on previous literature (Kawai et al., 2026); however, the optimal cutoff may vary across datasets. Future studies could consider data-driven threshold optimization methods to further reduce subjectivity and improve the generalizability of ICA-based denoising in fNIRS studies.

## 5 Conclusion

This study found that non-hand body-part images elicited stronger HbO responses than objects and hands within the sampled occipitotemporal cortex. The left and bilateral effects were significant in both the primary and ICA-preprocessed analyses, whereas the right-sided effects were significant only in the primary analysis. Overall, the findings support the feasibility of using occipitotemporal fNIRS to measure body-preferring responses consistent with EBA-related processing. However, they do not establish direct anatomical localization of the EBA, and extracerebral contributions cannot be fully excluded without short-separation measurements.

## Data Availability

The datasets presented in this study can be found in online repositories. The names of the repository/repositories and accession number(s) can be found below: The raw data supporting the findings of this study are openly available on Zenodo at DOI: 10.5281/zenodo.20542859.
