# Correction: EBA-related body-preferring responses in the occipitotemporal cortex measured with functional near-infrared spectroscopy

**DOI:** 10.3389/fnhum.2026.2022071

**Published:** 2026-10-19

**Authors:** Yuanchun Liu, Qiuya Zhao, Jiayu Wu, Ping Yang, Yuannong Ye, Min Wang

**Affiliations:** 1 School of Medical Humanities, Guizhou Medical University, Guiyang, China; 2 School of Biology and Engineering, Guizhou Medical University, Guiyang, China; 3 School of Psychology, Guizhou Normal University, Guiyang, Guizhou, China

**Keywords:** body-part selectivity, body-related visual processing, EBA, Extrastriate body area, fNIRS, occipitotemporal cortex

Author Min Wang was erroneously assigned to affiliation School of Psychology, Guizhou Normal University, Guiyang, Guizhou, China. This affiliation has now been removed for author Min Wang.

Affiliation School of Biology and Engineering, Guizhou Medical University, Guiyang, China was omitted for author Min Wang. This affiliation has now been added for author Min Wang.

The original version of this article has been updated.

