# Supplementary material for: EBA-related body-preferring responses in the occipitotemporal cortex measured with functional near-infrared spectroscopy
Source: Front Hum Neurosci. 2026 Sep 16;20:1914488. doi: 10.3389/fnhum.2026.1914488 (PMC13625679; doi:10.3389/fnhum.2026.1914488)
Supplement: Supplementary file 1 [file Table_1.DOCX]

**Supplementary Table S1.** MNI coordinates and probabilistic anatomical information for the channels included in the predefined occipitotemporal channel pools.

| Channel ID | Hemisphere | MNIx | MNIy | MNIz |
| --- | --- | --- | --- | --- |
| CH1 | Left | -52 | -76.33 | 35.33 |
| CH2 | Left | -39 | -93 | 23 |
| CH3 | Left | -60 | -66 | 28 |
| CH4 | Left | -48 | -86.33 | 16.67 |
| CH5 | Left | -28 | -103 | 6 |
| CH6 | Left | -54.67 | -77 | 10 |
| CH7 | Left | -39.67 | -94 | -1.67 |
| CH8 | Left | -61.33 | -65.67 | 1.67 |
| CH9 | Left | -45.33 | -83.33 | -10.33 |
| CH10 | Left | -51 | -70.33 | -16.67 |
| CH11 | Right | 41.67 | -88 | 22.67 |
| CH12 | Right | 56 | -66.33 | 38.33 |
| CH13 | Right | 33.67 | -99 | 2 |
| CH14 | Right | 52 | -78.67 | 19.67 |
| CH15 | Right | 64 | -55.67 | 31.33 |
| CH16 | Right | 45.33 | -89.67 | -0.33 |
| CH17 | Right | 59 | -69 | 13 |
| CH18 | Right | 53 | -78.33 | -6.33 |
| CH19 | Right | 67 | -56.33 | 4.67 |
| CH20 | Right | 59.67 | -66.33 | -12.67 |

*Note.* The coordinates were obtained from the pre-experimental montage-positioning test and characterize the expected cortical coverage of the standardized montage used in the formal experiment. They do not represent participant-specific channel coordinates for the formal sample or independent functional localization of the EBA.
